# Phospho-ERK and AKT status, but not *KRAS *mutation status, are associated with outcomes in rectal cancer treated with chemoradiotherapy

**DOI:** 10.1186/1748-717X-6-114

**Published:** 2011-09-12

**Authors:** Janine M Davies, Dimitri Trembath, Allison M Deal, William K Funkhouser, Benjamin F Calvo, Timothy Finnegan, Karen E Weck, Joel E Tepper, Bert H O'Neil

**Affiliations:** 1 Department of Medicine, Division of Hematology/Oncology, University of North Carolina at Chapel Hill, 170 Manning Dr, CB 7305, Chapel Hill, NC 27599-7305, USA; 2 UNC Lineberger Comprehensive Cancer Center, University of North Carolina at Chapel Hill, School of Medicine, CB 7295, Chapel Hill, NC, 27599-7295, USA; 3 Department of Pathology and Laboratory Medicine, University of North Carolina at Chapel Hill, Brinkhous-Bullitt Building, CB 7525, Chapel Hill, NC, 27599-7525, USA; 4 Lineberger Biostatistics Core, UNC Lineberger Comprehensive Cancer Center, University of North Carolina at Chapel Hill, School of Medicine, CB 7295, Chapel Hill, NC, 27599-7295, USA; 5 Department of Surgery, Division of Surgical Oncology, University of North Carolina at Chapel Hill, 170 Manning Dr, CB 7213, Chapel Hill, NC 27599-7213, USA; 6 Alamance Regional Medical Center, 1240 Huffman Mill Rd, Burlington, NC, 27215, USA; 7 Department of Radiation Oncology, University of North Carolina at Chapel Hill, 170 Manning Dr, CB 7305, Chapel Hill, NC 27599-7305, USA

**Keywords:** Rectal cancer, KRAS analysis, phosphoERK, phosphoAKT, radiation response

## Abstract

**Background:**

*KRAS *mutations may predict poor response to radiotherapy. Downstream events from *KRAS*, such as activation of *BRAF*, AKT and ERK, may also confer prognostic information but have not been tested in rectal cancer (RC). Our objective was to explore the relationships of *KRAS *and *BRAF *mutation status with p-AKT and p-ERK and outcomes in RC.

**Methods:**

Pre-radiotherapy RC tumor biopsies were evaluated. *KRAS *and *BRAF *mutations were assessed by pyrosequencing; p-AKT and p-ERK expression by immunohistochemistry.

**Results:**

Of 70 patients, mean age was 58; 36% stage II, 56% stage III, and 9% stage IV. Responses to neoadjuvant chemoradiotherapy: 64% limited, 19% major, and 17% pathologic complete response. 64% were *KRAS *WT, 95% were *BRAF *WT. High p-ERK levels were associated with improved OS but not for p-AKT. High levels of p-AKT and p-ERK expression were associated with better responses. *KRAS *WT correlated with lower p-AKT expression but not p-ERK expression. No differences in OS, residual disease, or tumor downstaging were detected by *KRAS *status.

**Conclusions:**

*KRAS *mutation was not associated with lesser response to chemoradiotherapy or worse OS. High p-ERK expression was associated with better OS and response. Higher p-AKT expression was correlated with better response but not OS.

## Background

The most common genetic mutation in all cancers is the *RAS *mutation, occurring in 30% of cancers [1]. The RAS family of genes (*K-, H-*, and *N-RAS*) transduce, and likely integrate, messages from growth factor receptors [2]. Intracellular pathway signaling via the *RAS *pathway causes cell proliferation and invasion along with evasion of apoptosis [2,3]. In rectal cancer (RC) series that included patients with stage IV disease, *KRAS *mutations, are reported in 19 to 48% of patients[4-6]. This is similar to metastatic colorectal cancer (mCRC) in which mutations are found in 30 to 40% of patients, mostly at codons 12, 13, and 61. Mutation of these codons of *KRAS *leads to constitutive activation of the RAS signaling pathway. There are conflicting data with regards to the predictive and prognostic significance of *KRAS *mutations in colon cancer, and less is known for RC. However, in an initial assessment of *RAS *oncogene expression in RC, one retrospective study evaluated the RAS gene protein product, p21 [7]. Higher p21 titers were associated with worse 5 year survival (44 vs. 64%, p < 0.02), more frequent distant metastases (52 vs. 23%, p < 0.001), and more advanced stage (54 vs. 36% incidence of Dukes' C, p < 0.04) [7].

*RAF *is one of several targets of activated *RAS*, and itself is mutated in approximately 8 to 10% of mCRC,[8] and 0 to 12% specifically in RC [5,6,9,10]. There is strong evidence to suggest that *KRAS *and *BRAF *mutations are mutually exclusive [8,11-13]. Constitutive activation of *BRAF *leads to extracellular signal-regulated kinase (ERK) phosphorylation (p-ERK) and activation [14]. ERK is required for cell proliferation and allows for the evasion of apoptosis [14]. To date, only small studies have been reported in mCRC, but these suggest that outcomes are worse among patients with *BRAF *mutations [8,13]. In RC, this has not been corroborated and the effect(s) of ERK has not been elucidated.

The phosphatidylinositol 3-kinase (PI3K)/AKT pathway is another downstream activation target of mutated *KRAS*, and is important in cell metabolism, growth, motility, survival, proliferation, and metastasis [15-18]. Following activation through receptor kinases or activated *RAS*, PI3K phosphorylates and activates AKT (pAKT) [19]. Activation of AKT by PI3K is inhibited by the tumor suppressor gene *PTEN*,[19] which is occasionally lost via mutation or gene silencing in CRC [20,21]. In small cell lung cancer, elevated phosphorylated AKT was associated with limited stage disease but was not prognostic [22]. In prostate cancer, AKT expression intensity has been associated with serum levels of prostate specific antigen [23]. In breast cancer, p-AKT expression was inversely correlated with survival [24]. Ionizing and UV radiation are known to activate the PI3K/AKT pathway [25]. Activating mutations of the PI3K p110 subunit are found in 10 to 30% of colon cancers [17,26,27]. In mCRC, it is not clear if mutation of PI3K or activation of the pathway has an impact on survival or other outcomes [19,26].

Based on the above preclinical and clinical findings, the objective of this study was to explore the relationships of *KRAS *and *BRAF *status with p-AKT and p-ERK expression and with clinical outcomes (response to radiation and overall survival [OS]) in rectal adenocarcinoma. Many downstream targets of RAS can be inhibited by small molecule agents, yet potential consequences of such inhibition are not known. The principal rationale was to determine which RAS-activated pathways are relevant to radiation (RT) resistance in order to select appropriate targets for therapy. Both *KRAS *and *BRAF *mutations were hypothesized to confer radioresistance and result in lesser response to RT and worse survival. Activation of either AKT or ERK was also hypothesized to antagonize chemoradiotherapy effects, be associated with worse survival, and be associated with mutations of *KRAS *or *BRAF*.

## Methods

This was a retrospective study of RC patients identified through the Institutional Tumor Registry, who received preoperative chemoradiotherapy with concurrent 5FU chemotherapy followed by surgical resection, and had adequate tissue for evaluation in the preoperative biopsy and/or surgical resection sample. Clinical data were collected from the patients' medical records with complete follow-up until April 23, 2008, at which point survival data were censored. Conduct of the study was performed with approval by our Institutional Review Board and in accordance with the Helsinki Declaration of 1975, as revised in 2000.

The primary clinical outcome measure for this study was pathologic response, which was divided into three objective categories: limited response (pLR, gross residual disease present), major response (pMR, only microscopically visible disease remaining), or complete response (pCR, no pathologic evidence of residual tumor cells). AJCC pathologic stage at surgery was also assessed and compared with preoperative staging to determine treatment response (downstaging).

### Mutation analysis

*KRAS *and *BRAF *mutation testing were performed by pyrosequencing as described elsewhere,[28-31] using preoperative or postoperative samples. All samples were enriched for tumor cells by microdissection prior to mutation testing. An H&E stained slide prepared from formalin-fixed paraffin embedded tissue samples was examined under a light microscope by a pathologist and an area containing at least 50% tumor cells was microdissected from adjacent unstained slides for tumor enrichment prior to xylene deparaffinization and DNA extraction. DNA was extracted using the Qiagen DNeasy Tissue Extraction Kit from tissue samples pretreated with xylene for removal of paraffin. Pyrosequencing was performed to identify *KRAS *mutations in codons 12, 13 and 61 and the *BRAF *V600E mutation using the PyroMark Q96 *KRAS *v2.0 kit (#972452, Qiagen) and PyroMark *BRAF *RUO Kit (#40-0057, Qiagen), respectively, as per the manufacturer instructions. PCR reactions were performed on a Veriti thermal cycler (Applied Biosystems) and pyrosequencing was performed on the PyroMark MD (Pyrosequencing AB). A positive control, normal control, and blank (no DNA template) PCR control were included in each assay. Pyrograms were analyzed by PyroMark 1.0 software using allele quantification (AQ) mode to determine the percentage of mutant versus wild-type alleles according to relative peak height.

### Immunohistochemistry (IHC)

was performed on preoperative biopsies using previously published methods [32]. Briefly, unstained 5-micron-thick sections were baked at 60°C for 15 to 60 minutes. Baked sections were soaked twice in fresh xylene for 5 minutes each, then soaked in 100% ethanol for 3 minutes, and blocked for endogenous peroxidase with 3% hydrogen peroxide in methanol for 10 minutes. Slides were soaked in 95% ethanol for 3 minutes, 70% ethanol for 3 minutes, rinsed in distilled water and soaked in Dako wash buffer (Dako Cat. No. S3006; Dako, Glostrup, Denmark) for 5 minutes. Slides were then steamed in a Black & Decker steamer for 25 minutes using antigen retrieval buffers (Dako) for each primary antibody to be studied (Ser-473 antibody for p-AKT, Cell Signaling Systems, Cat. No. 736E11; Thr-202/Tyr-204 antibody for p-ERK, Cell Signaling Systems, Ca. No. D13.14.4E) and then allowed to cool for at least 20 minutes. Sections were transferred to Dako wash buffer for 5 minutes. Endogenous biotin was neutralized by incubating the slides in a biotin blocking system (Dako Cat. No. X0590) for 10 minutes at room temperature in each of the 2 solutions. Sections were then exposed to the primary antibodies (Ser-473 antibody at 1:25 dilution; Thr-202/Tyr-204 antibody at 1:200 dilution) for 30 minutes at room temperature. After rinsing in Dako wash buffer, slides were incubated with the Dako LSAB2 biotinylated link for 10 minutes at room temperature, rinsed in Dako wash buffer, and then incubated with the Dako LSAB2 streptavidin-horseradish peroxidase for 10 minutes at room temperature. After additional rinsing in Dako wash buffer, detection of the antibody/antigen complex was visualized using 3-3 diaminobenzidine for 5 minutes. Slides were then rinsed in water, lightly counterstained in filtered Mayer's hematoxylin, rinsed, dehydrated, cleared, and mounted. The cells of interest (tumor cells) in each section were scored for percentage reactivity and signal strength in both the cytoplasm and the nuclei. Simultaneously stained normal rectal mucosa and no primary antibody stained normal mucosa served as negative controls in each experiment.

Nuclear peroxidase staining (chosen because it was more consistent from sample to sample than cytoplasmic staining) was scored by one surgical pathologist (WKF) who was blinded to clinical information. Sections were scored by multiplying the average staining intensity seen on a scale of 0 to 3+ by the percentage of cells that were positive to any degree, creating a range of possible scores of 0 to 300.

### Statistical Methods

Categorical data were analyzed using Fisher's Exact Test. Continuous data comparing pathological response and stage with p-AKT and p-ERK activation used Jonckheere-Terpstra tests to account for ordered differences among groups, and Wilcoxon Rank Sum tests were used to compare p-AKT and p-ERK among mutation groups. Survival curves were created using the Kaplan Meier method and were compared using the Log rank test; OS was calculated from the time of radiation treatment. Cox regression analyses were used to explore the association of p-AKT and p-ERK activation with OS. Analyses were performed using SAS v9.2 statistical software.

## Results

Patient characteristics are summarized in Table 1 (n = 70). Six patients (9%) had stage IV disease at diagnosis for which initial treatment with chemoradiotherapy was selected. At the time of surgery, 43% of patients were downstaged and 17% had a pCR. All but two patients received concurrent 5-FU chemotherapy with the radiation. There was no association of age, sex, nor race with stage at diagnosis (all p > 0.2).

**Table 1:** Patient characteristics and clinical data

Characteristics		N = 70
Age at diagnosis	Median	58 years
	Range	26-89
Gender	Male	42 (60%)
	Female	28 (40%)
Race	White	52 (74%)
	Black	15 (21%)
	Other or unknown	3 (4%)
Clinical disease stage (at diagnosis)	II	25 (36%)
	III	39 (56%)
	IV	6 (9%)
Pathological disease response	Complete response (pCR)	12 (17%)
	Major response (pMR)	13 (19%)
	Limited response (pLR)	45 (64%)
Treatment response	Downstaged	30 (43%)
	No change	33(47%)
	Upstaged	7 (10%)
Recurrence	No	45 (64%)
	Yes	25 (36%)
	Local recurrence	7 (10%)
	Distant recurrence	13 (19%)
	Both local and distant	5 (7%)
Status (censored April 23, 2008)	Alive	41 (59%)
	Dead	29 (41%)

pCR: Complete response; pMR: Major response; pLR: Limited response

With a median follow-up for survivors of 42 months, 36% experienced recurrence, 72% of which were distant recurrences. For all patients, the median OS was 4.5 years (95% CI 3.0-12.5).

*KRAS *and *BRAF *mutation testing was successfully performed on 67 and 64 of the tumors, respectively. Among evaluable samples, 24 (36%) were mutant for *KRAS *and 3 (5%) were mutant for *BRAF *(Table 2). *KRAS *and *BRAF *mutations were mutually exclusive except in one patient (who had a pCR and therefore further tissue was not available) in which the tumor was positive for both *KRAS *and *BRAF *mutations.

**Table 2:** *KRAS *and *BRAF *mutational status with outcomes

	*KRAS (n = 67)*	*BRAF (n = 64)*
Wild-type (WT)	43 (64%)	61 (95%)
Mutant	24 (36%)	3 (5%)
Median OS WT	4.1 years	4.1 years
Median OS mutant	4.9 years p = 0.6	Not reached p = 0.1
WT vs. mutant		
Limited response	67% vs. 67%	69% vs. 33% (1 of 3)
Major response	14% vs. 21%	16% vs. 0%
Complete response	19% vs. 13% p = 0.7	15% vs. 66% (2 of 3)
WT vs. mutant		
Downstaged	42% vs. 38%	36% vs. 66% (2 of 3)
No change or upstaged	58% vs. 63% p = 0.8	64% vs. 33% (1 of 3)
Recurrence		
None	67% vs. 54%	62% vs. 100% (3 of 3)
Local recurrence	12% vs. 8%	10%
Distant recurrence	14% vs. 29%	20%
Both local and distant	7% vs. 8% p = 0.5	8%

WT: Wildtype; OS: Overall Survival

### *KRAS/BRAF *mutations do not predict chemoradiation response in rectal cancer

One of our principal hypotheses based on preclinical information was that *KRAS *mutation would confer radioresistance and result in reduced response to RT. Our results, however, did not confirm this hypothesis (Table 2). There was no difference in residual tumor status, difference in radiation response as assessed by downstaging of tumors, or frequency of local recurrence based on *KRAS *or *BRAF *status.

### *KRAS/BRAF *mutations do not predict overall survival in rectal cancer

There was no difference in OS by *KRAS *mutation status (median OS 4.1 years for WT vs. 4.9 years for mutated tumors, p = 0.6, Table 2), consistent with recent results in mCRC [33]. The number of patients with *BRAF *mutation was small and we did not find a significant difference in OS by *BRAF *mutation status (median OS 4.1 years for WT vs. not reached for mutated tumors, p = 0.1). Interestingly, none of the three *BRAF *mutant patients died during follow-up, a finding that is limited given the small number of patients.

### AKT activation status, but not ERK activation status, correlates with *KRAS *mutation status

**Figure 1:**
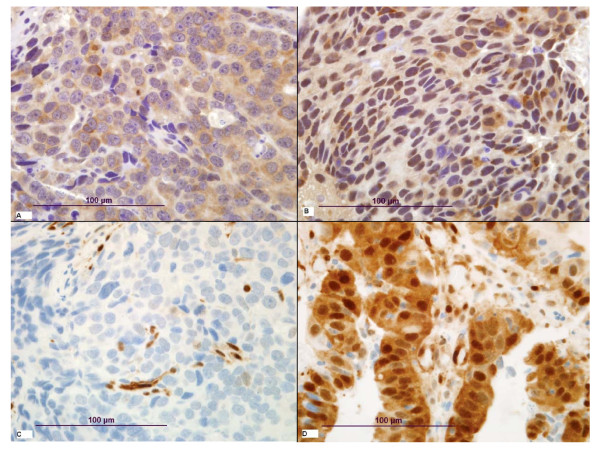
**Representative examples of immunohistochemistry staining intensity for p-AKT (low intensity, panel A; high intensity, panel B) and p-ERK (low intensity, panel C; high intensity, panel D) at 60× magnification**.

**Figure 2:**
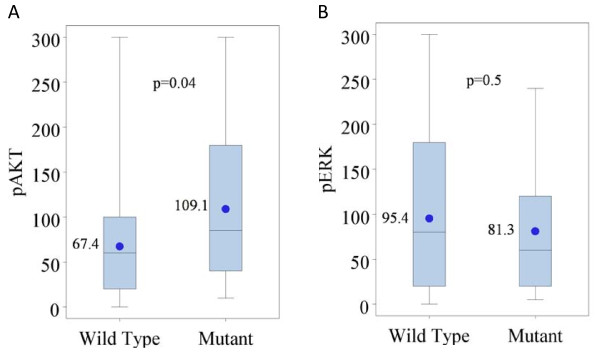
**p-AKT (A; wildtype n = 41, mutant n = 24) and p-ERK (B; wildtype n = 42, mutant n = 23) expression by *KRAS *mutational status**.

Because ERK and AKT are both potential targets of activated/mutated *KRAS*, we explored correlations between *KRAS *mutation and phosphorylation status of these molecules. Representative examples of low and high staining intensity are demonstrated for pAKT and pERK (Figure 1). Median nuclear p-AKT staining intensity was 80 (range 0-300). Patients with mutant *KRAS *tumors had higher p-AKT scores (mean = 109) than *KRAS *WT tumors (mean = 67, p = 0.04, Figure 2A). Median nuclear p-ERK staining intensity was 80 (range 0-300). Surprisingly, and in contrast to p-AKT, there was no difference noted in p-ERK intensity by *KRAS *mutation status (p = 0.5, Figure 2B). No significant association was noted between p-ERK levels and *BRAF *mutational status in this small group of patients.

### p-AKT and p-ERK activation are associated with better response to chemoradiotherapy

**Figure 3:**
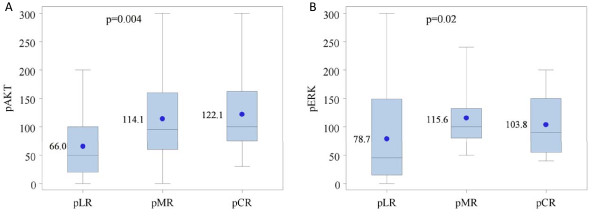
**p-AKT (A; pLR n = 43, pMR n = 13, pCR n = 12) and p-ERK (B; pLR n = 44, pMR n = 12, pCR n = 12) expression by radiation response (residual disease at the time of surgery) with responses categorized as limited response (pLR), major response (pMR), or complete response (pCR)**.

Based on existing preclinical information, we hypothesized that activation of either AKT or ERK would antagonize chemoradiotherapy effect based on inhibition of apoptosis [34,35]. To test this, we examined associations between p-AKT or p-ERK activation status defined by IHC with chemoradiotherapy response and survival. Our results stand in contradiction to our hypothesis, however, in that increasing intensity of both p-AKT and p-ERK were associated with less residual disease suggestive of better responses to RT (Figure 3). Patients with pCR or pMR at the time of surgery had higher p-AKT levels than those with pLR (p = 0.006). When the three pathologic response categories were compared by p-ERK levels, differences were noted (p = 0.02). p-ERK levels were significantly higher in the pMR group, but not in the pCR group, when compared to the pLR (p = 0.03, 0.07 respectively).

### p-ERK activation is associated with survival, but not p-AKT

**Figure 4:**
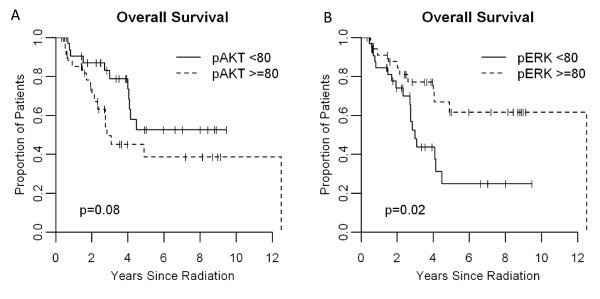
**Overall survival by p-AKT (A; p-AKT < 80, n = 33, p-AKT ≥80, n = 35) and p-ERK (B; p-ERK < 80, n = 32, p-ERK ≥80 n = 36) expression**.

We lastly hypothesized that either p-ERK or p-AKT activation would be associated with worse survival. On the contrary, patients with tumor p-ERK expression above the median had significantly longer survival compared with those below (12.5 vs. 3.0 years, p = 0.016, Figure 4B). This survival difference was maintained but lost statistical significance when the stage IV patients were excluded from the analysis (p = 0.06). Overall, for each 50 point decrease in p-ERK staining intensity, the risk of death increased by 32% (p = 0.06), reflecting a benefit to increased p-ERK levels. A borderline significant improvement in OS was also detected with lesser staining intensity of p-AKT (p = 0.08, Figure 4A). When the stage IV patients were excluded, patients with tumor p-AKT expression below the median had significantly longer survival than those below the median (p = 0.029).

## Discussion

In RC patients treated with chemoradiotherapy, *KRAS *mutation was not associated with lesser response to chemoradiotherapy or worse OS. While both p-AKT and p-ERK expression were correlated with better response to chemoradiotherapy, only high p-ERK expression was associated with better OS.

Constitutive activation of *KRAS *via mutation is a common finding in CRC, and one that has been assumed to be of importance to tumorigenesis due to its frequent and early occurrence in the adenoma-carcinoma sequence. Recent studies in mCRC have demonstrated that responses to epidermal growth factor receptor (EGFR) inhibitors (cetuximab or panitumumab) occur only in patients with the wild-type (WT) *KRAS *gene. These drugs are now used in the treatment of mCRC, with response rates ranging from 8% with single agent cetuximab[36] to 23% in combination with irinotecan,[37] and disease control rates (a combination of complete and partial responses and stable disease) of 39 and 56% respectively.

Preclinical studies have suggested that RAS mutation would lead to radioresistance, and conversely that targeting RAS or downstream effectors of activated *KRAS *would identify potential tumor-specific radiosensitizers [38-40]. This in part formed the rationale for targeting of EGFR in RC, an intervention that has to date been disappointing in clinical trials. Studies of RAS in human RC tumor samples have generally been small and have produced mixed conclusions [2,5,7,41-43]. These studies have reported no change in downstaging[41] or disease free status by *KRAS *status [5]. Other studies found that *KRAS *WT was associated with improved survival,[2] or trended to tumor downstaging[7] or response,[42] while another study found associations between *KRAS *mutation with earlier stage and better survival [43]. Our study set out to confirm that *KRAS *mutational status was associated with radiosensitivity using more modern sequencing technology in a larger number of patients than had been studied previously. Our findings did not confirm *KRAS *mutation as a prognostic factor, nor as a predictive factor for resistance to chemoradiotherapy, suggesting that *KRAS *may not be a worthwhile target to pursue as a radiosensitizer in RC.

Our findings confirm a relatively low rate of *BRAF *mutation as is seen in mCRC, with numbers too small to draw conclusions about the effect of *BRAF *on chemoradiotherapy response. However, we note that all three patients with confirmed *BRAF *mutations were long-term survivors.

This study also represented an opportunity to assess the effects of *KRAS *mutation on activation of its downstream targets ERK and AKT. Mutation or activation of *KRAS *results in constitutive activation of several downstream effectors, and to date it has not been clear which effectors are most important in mediating the effects of mutated *KRAS *in cancer cells [1]. We found that AKT activation was significantly associated with *KRAS *activation, while ERK activation was not. One potential weakness of this finding, however, is that anti-phosphoprotein antibodies were used in paraffin-embedded tissues of various ages with the possibility that there was variability in the stability of the phosphorylated proteins. Another small study of predominantly stage II and III CRC tumors evaluated the *KRAS/BRAF*/ERK pathway. p-ERK was correlated with *KRAS *codon 12 mutations (p = 0.016) but not with codon 13 mutations [14]. *BRAF *mutations (V599) were infrequent (8.7%) and not associated with p-ERK status [14]. We did not examine the association between mutation location and p-ERK in our study. Preoperative sample limitations prevented all phosphorylation and mutational analyses to be conducted on these untreated samples.

Among our most interesting findings was that higher p-ERK levels were associated with better survival and response to chemoradiotherapy, and that higher p-AKT expression was also associated with better response to chemoradiotherapy. Certain tumors exhibiting activated AKT, including squamous cell cancer of the head and neck[44] and cervical cancer,[34] have been shown to respond poorly to chemoradiotherapy. The positive association between ERK activation and response is less surprising, as ERK activation results in increased cell cycling,[45] and presence of higher fractions of cycling cells has previously been associated with better outcome from chemoradiotherapy for RC [46]. The positive association between ERK activation and survival is the first described in RC to our knowledge. In hepatocellular carcinoma, higher pERK staining intensity was associated with longer time to progression [47].

It is more difficult to understand why AKT activation would be beneficial in terms of response. Data from other tumor types suggests that activation of the AKT cell survival pathway confers resistance to radiation [34,44]. For example, in a PTEN-deficient glioma model, a case in which AKT is constitutively active, PTEN gene transfer resulted in significant radiosensitization [48]. In a recent publication, it was demonstrated that PI3K mutations, which would be expected to result in constitutive AKT activation, were associated with a higher rate of local recurrence of RC (27.8 vs. 9.4%) [6].

## Conclusions

In summary, we assessed RAS and selected downstream effectors in order to determine the relationships between *KRAS *(and *BRAF*) with these effectors and clinical outcomes. Our results suggest that activation of AKT and ERK may be beneficial for response to radiation therapy, and thus targeting these pathways in the setting of chemoradiotherapy could possibly be contraindicated and should be evaluated prospectively with a sufficient sample size.

## List of Abbreviations

ERK: extracellular signal-regulated kinase; IHC: immunohistochemistry; mCRC: metastatic colorectal cancer; OS: overall survival; p-AKT phosphorylated AKT; pCR: pathologic complete response; p-ERK: phosphorylated extracellular signal-regulated kinase; PI3K: phosphatidylinositol 3-kinase (AKT); pLR: pathologic limited response; pMR: pathologic major response; RC: rectal cancer; RT: radiation

## Competing interests

The authors declare that they have no competing interests.

## Authors' contributions

BHO conceived the study and design, coordinated the study, prepared IHC samples, participated in the data analysis, interpretation of results, and drafted the manuscript. KW and DT carried out the molecular genetic studies. KW participated in the data interpretation. WKF carried out the immunohistochemical studies. TF participated in clinical data collection. AMD performed the statistical analyses. JMD participated in the data analyses, interpretation of results, and drafted the manuscript. BFC participated in the study development and data analysis. JET participated in the study design, data interpretation and manuscript revisions. All authors read and approved the final manuscript.
